# Transcriptome analysis of various flower and silique development stages indicates a set of class III peroxidase genes potentially involved in pod shattering in *Arabidopsis thaliana*

**DOI:** 10.1186/1471-2164-11-528

**Published:** 2010-09-29

**Authors:** Claudia Cosio, Christophe Dunand

**Affiliations:** 1 Institut Forel, University of Geneva, 10 route de Suisse, CP416, CH-1290 Versoix, Switzerland; 2 SCSV-UMR5546 CNRS/UPS, 24 Chemin de Borderouge, BP 42617, 31326 Castanet-Tolosan, France

## Abstract

**Background:**

Plant class III peroxidases exist as a large multigenic family involved in numerous functions suggesting a functional specialization of each gene. However, few genes have been linked with a specific function. Consequently total peroxidase activity is still used in numerous studies although its relevance is questionable. Transcriptome analysis seems to be a promising tool to overcome the difficulties associated with the study of this family. Nevertheless available microarrays are not completely reliable for this purpose. We therefore used a macroarray dedicated to the 73 class III peroxidase genes of *A. thaliana *to identify genes potentially involved in flower and fruit development.

**Results:**

The observed increase of total peroxidase activity during development was actually correlated with the induction of only a few class III peroxidase genes which supports the existence of a functional specialization of these proteins. We identified peroxidase genes that are predominantly expressed in one development stage and are probable components of the complex gene networks involved in the reproductive phase. An attempt has been made to gain insight into plausible functions of these genes by collecting and analyzing the expression data of different studies in plants. Peroxidase activity was additionally observed *in situ *in the silique dehiscence zone known to be involved in pod shattering. Because treatment with a peroxidase inhibitor delayed pod shattering, we subsequently studied mutants of transcription factors (TF) controlling this mechanism. Three peroxidases genes -*AtPrx13*, *AtPrx30 *and *AtPrx55- *were altered by the TFs involved in pod shatter.

**Conclusions:**

Our data illustrated the problems caused by linking only an increase in total peroxidase activity to any specific development stage or function. The activity or involvement of specific class III peroxidase genes needs to be assessed. Several genes identified in our study had not been linked to any particular development stage or function until now. Notably *AtPrx13*, which is one of the peroxidase genes not present on commercially available microarrays. A systematic survey of class III peroxidase genes expression is necessary to reveal specific class III peroxidase gene functions and the regulation and evolution of this key multifunctional enzyme family. The approach used in this study highlights key individual genes that merit further investigation.

## Background

Genes encoding secreted class III plant peroxidases (EC 1.11.1.7) are present in all land plants and form large multigenic families [1]. In their regular peroxidative cycle, class III peroxidases catalyze the reduction of H_2_O_2 _by taking electrons to various donor molecules [2]. An hydroxylic cycle, which leads to the formation of various radical species such as ^·^OH or HOO^·^, has also been described [1]. Plant peroxidases are involved in a broad range of physiological processes throughout the plant life cycle [3], such as the formation of phenolic polymers as well as auxin catabolism [4-6]. The great number of plant peroxidases genes, the diversity of the processes catalyzed by them, as well as the presence of both highly conserved domains and variable parts in all their sequences suggest the existence of a functional specialization of these proteins [7]. It is therefore imperative to link each individual gene with a precise role for a better understanding of the functions, the regulation and also the evolution of this key multifunctional enzyme family.

In an attempt to identify the function of specific class III peroxidases, several authors reported the generation of transgenic plants to study different peroxidase genes. However, in *A. thaliana *only 9 out of 73 peroxidase genes have been identified by this approach (Table 1). The characterisation of individual peroxidase mutants often gives only unconclusive results [8-12]. The *in planta *role of most peroxidases remains therefore elusive. This situation is mainly linked to two difficulties inherent in peroxidases: i) gene redundancy results in no visible mutant phenotype, and ii) the lack of substrate specificity *in vitro*, prohibits a determination of which compounds are real *in planta *substrate. Molecular biology approaches seems to offer a powerful tool to overcome these difficulties [7]. Transcriptome analysis for example provides a detailed description of the gene regulation during plant growth and development, in any plant tissue and also in different relevant genotypes. Thus a transcriptomic approach should permit an efficient screen showing which peroxidases are expressed at key developmental stages, but also to identify redundant peroxidases genes putatively involved in the same specific process in all kind of tissues, development stages and genotypes. Nevertheless, not all peroxidase genes are represented on commercially available microarrays. For this reason, in this study we used a home made macroarray dedicated exclusively to the 73 class III peroxidase genes of *A. thaliana *[13].

**Table 1:** List of *Arabidopsis thaliana *class III peroxidases genes putatively involved in a specific mechanism identified by transgenic plant approaches

Protein name	Organ	Mechanism of interest of the study	References
**AtPrx03**	roots	cold inducible tolerance	[57]
**AtPrx17**	flowers	silique lignification	[7]
**AtPrx21**	leaves	fungus defense	[59]
**AtPrx33**	leaves	oxidative burst	[35]
	roots	root length	[74]
**AtPrx34**	leaves	oxidative burst	[35]
	roots	root length	[74]
**AtPrx53**	whole plant	lignification of vascular bundles	[55]
**AtPrx62**	leaves	fungus defense	[59]
**AtPrx66**	roots	lignification of vascular bundles	[75]
**AtPrx71**	leaves	fungus defense	[59]

The aim of the present study was to identify class III peroxidase genes involved in flower and fruit development in *A. thaliana*. We conducted a macroarray analysis of the 73 class III peroxidase genes over 5 different development stages from flower buds to senescing siliques. These data were compared to class III peroxidase gene expression in leaves as well as combined with histological observations. Peroxidase activity was detected *in situ *in endocarp (en*b*) with heavy lignification associated with a simultaneous reduced cell-to-cell cohesion of the dehiscence zone (DZ) which is believed to be responsible for pod shattering [14,15]. In the present study, we further showed in *A. thaliana *that when class III peroxidase activity was inhibited pod shattering was delayed. We therefore analysed peroxidase gene expression in different plant lines known to be affected in pod shattering and identified three putative class III peroxidase genes whose expression was altered therein. Pod shatter is a well studied mechanism that is responsible for seed dispersal in *Brassicacea *including *A. thaliana *[16]. The vast majority of the genes identified to date in the process encode transcription factors involved in the regulation of gene expression. In contrast, comparatively few downstream target genes have been identified that are involved in organ formation. To our knowledge, no specific peroxidase gene has been shown to be involved in dry fruit maturation until now.

## Results

### Total peroxidase activity increases during development and senescence

**Figure 1:**
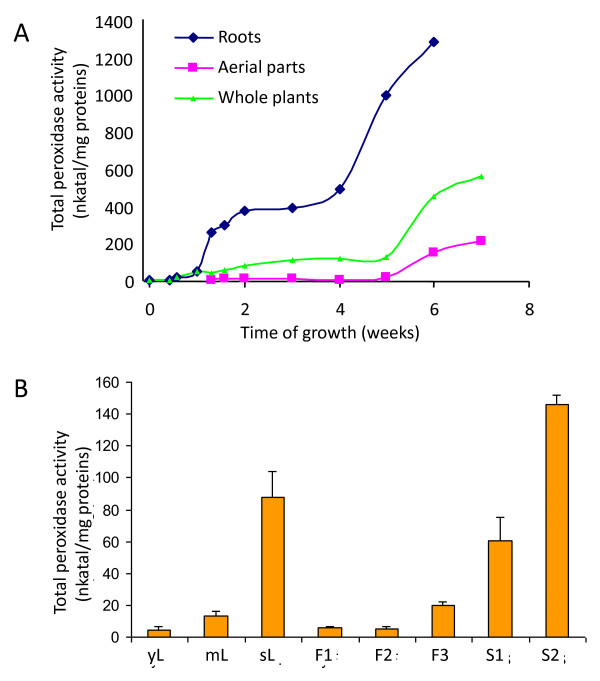
**Total peroxidase activity increases during *Arabidopsis thaliana *development**. Total peroxidase activity from germination to 7 week-old plants in roots, whole plants and aerial parts (A). Total peroxidase activity in various leaves, flower and siliques developmental stages (B). Young leaves (yL), mature leaves (mL), senescing leaves (sL), flower buds (F1 = stage 1-12), mature flowers (F2 = stage 13-14), senescing flowers (F3 = stage 15-16), mature siliques (S1 = stage 17), and senescing siliques (S2 = stage 18). (n = 3).

Class III peroxidase activity was higher in roots than aerial parts and increased with the age of the plant (Figure 1A). After the 5^th ^week of growth, an increase of peroxidase activity coincided with bolting. In the aerial part, peroxidase activity also increased with the age of the organ (Figure 1B). Indeed, senescent leaves showed a 13-fold larger peroxidase activity than mature leaves and a 25-fold higher activity than young leaves. In flowers and siliques, a similar evolution of the total peroxidase activity was observed. Senescent siliques (S2) showed a 2.4-fold higher activity than mature siliques (S1) and a 7.3-fold larger activity than senescing flowers (F3).

### Anthocyanin, chlorophyll and lignin content during development and senescence

**Figure 2:**
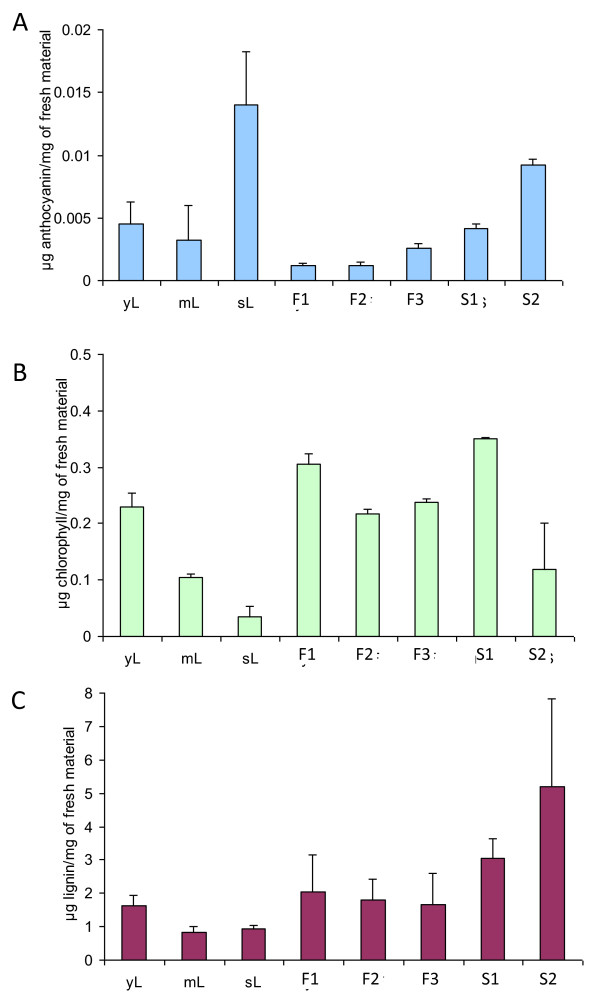
**Chlorophyll (A), anthocyanin (B) and lignin (C) contents in various leaf, flower and silique development stages in *Arabidopsis thaliana***. Young leaves (yL), mature leaves (mL), senescing leaves (sL), flower buds (F1), mature flowers (F2), senescing flowers (F3), mature siliques (S1), and senescing siliques (S2).

Chlorophyll loss, as well as anthocyanin and lignin increases, during development has been widely reported. These are known mechanisms associated with senescence and maturation that seem to be related with the increase in total peroxidase activity. In accordance with the literature, anthocyanin accumulation increased in senescing aerial organs (Figure 2A): senescing flowers showed 4.4-fold larger anthocyanin content than buds, whereas senescing siliques contained 10.9-fold larger anthocyanin than mature siliques. Chlorophyll content decreased with the age of organs (Figure 2B): young leaves contained 1.8-fold and 3.9-fold more chlorophyll than mature leaves and senescing leaves respectively. In flowers and S1 siliques chlorophyll content was relatively stable showing no significant differences. In contrast, S1 siliques contained 3.1-fold more chlorophyll than senescing siliques. Lignin deposition increased over time in siliques but not in leaves or flowers (Figure 2C): in senescing siliques we monitored 1.4-fold and 2.3-fold larger lignin content than respectively in S1 siliques and senescing flowers.

It is probable that class III peroxidases have a role in the above developmental mechanisms, as widely admitted in the scientific community, but the link between these phenotypical observations and total peroxidase activity is rather indirect. We therefore wanted to assess changes in expression of class III peroxidases to indicate candidate genes for such processes. Gene expression data was then verified by observing peroxidase isoform pattern at the same developmental stages.

### The increase of total peroxidase activity observed during development of flower and siliques is due to expression of only a few genes

**Figure 3:**
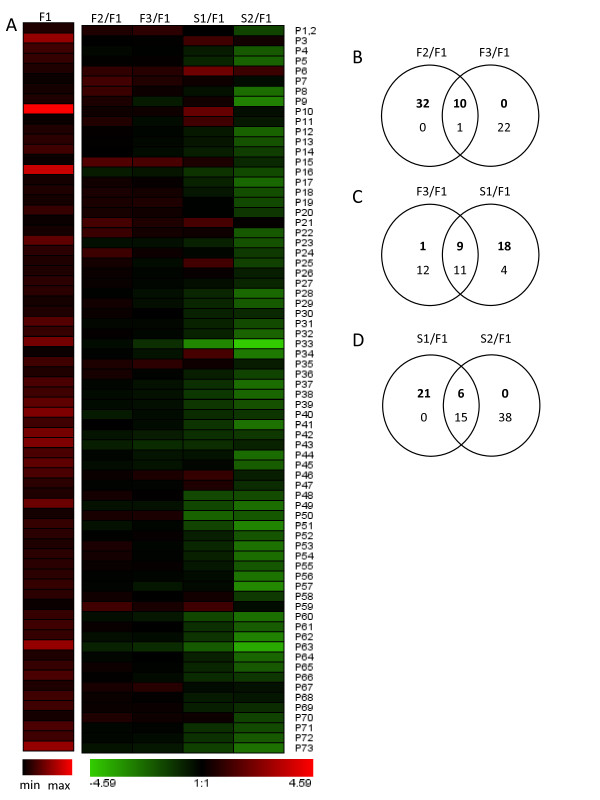
**Peroxidase expression profile during flower and silique development at the gene level**. (A). Macroarrays of flowers and siliques various stages (A). In the first column expression levels of each gene are represented as a ratio to the expression level of histone constitutively expressed gene (n = 3). In other columns, expression of peroxidase genes is represented as a ratio to their own expression level in F1 stage in log scale (A). Venn diagram illustrating the number of class III peroxidase genes up- (top and bold number) or down- (bottom number) regulated during various development stages when compared to F1 stage in *A. thaliana *(B-D) (F1 = stage 1-12, F2 = stage 13-14, F3 = stage 15-16, S1 = stage 17, S2 = stage 18). Only genes showing a differential expression of at least 1.3 up or 0.7 down and a significant Student t-test score (p < 0.05) were included in Venn diagrams (B-D).

The specific expression of the 73 peroxidase genes were observed using macroarrays in the various development stages of flowers and siliques (Figure 3A, Table 2, Table 3). Results were validated by semi-quantitative RT-PCR.

**Table 2:** Peroxidase genes the most highly and lowly expressed in the F1 stage in macroarray analysis as fold-difference to the histone reference gene expression level

gene	Expression level	gene	Expression level
*AtPrx10*	2.37 ± 0.01	*AtPrx11*	0.05 ± 0.003
*AtPrx16*	1.94 ± 0.004	*AtPrx34*	0.006 ± 0.001
*AtPrx03*	1.38 ± 0.053	*AtPrx15*	0.008 ± 0.002
*AtPrx73*	1.35 ± 0.049	*AtPrx21*	0.009 ± 0.003
*AtPrx63*	1.41 ± 0.014	*AtPrx59*	0.014 ± 0.007
*AtPrx40*	1.19 ± 0.06	*AtPrx07*	0.014 ± 0.002

**Table 3:** Expression level changes of peroxidase genes in macroarrays during flower and silique development

Highest in F1	Highest in F2	Highest in S1	Highest in F1-F2	Highest in F1-F3	Highest in S1-S2	Highest in F2-S1	Stable F1-S2
*AtPrx16*	*AtPrx7*	*AtPrx10*	*AtPrx23*	*AtPrx48*	*AtPrx3*	*AtPrx1,2*	*AtPrx26*
*AtPrx43*	*AtPrx8*	*AtPrx11*	*AtPrx33*	*AtPrx50*	*AtPrx6*	*AtPrx15*	*AtPrx27*
*AtPrx73*	*AtPrx24*	*AtPrx25*	*AtPrx37*	*AtPrx51*	*AtPrx7*	*AtPrx20*	*AtPrx30*
	*AtPrx29*	*AtPrx34*	*AtPrx38*			*AtPrx21*	*AtPrx36*
	*AtPrx36*	*AtPrx45*	*AtPrx41*			*AtPrx22*	*AtPrx40*
	*AtPrx48*	*AtPrx46*	*AtPrx45*			*AtPrx35*	*AtPrx42*
	*AtPrx53*	*AtPrx47*	*AtPrx49*			*AtPrx59*	*AtPrx43*
	*AtPrx55*		*AtPrx60*				*AtPrx66*
	*AtPrx58*		*AtPrx61*				*AtPrx68*
	*AtPrx65*		*AtPrx63*				*AtPrx71*
	*AtPrx69*						

In fully opened flowers (F2) 42 genes were up-regulated, one was down-regulated and 30 genes had a relatively stable level of expression when compared to F1. In senescing flowers (F3) 10 genes were up-regulated, 23 genes down-regulated and 40 genes had relatively stable level of expression when compared to F1. In senescing siliques, only six genes were up-regulated, 52 genes were down-regulated and 15 genes had relatively stable level of expression when compared to F1. These data therefore indicated a general lower expression level of the vast majority of peroxidase genes in older development stages with the exception of stage S1. In the same line, the Venn diagram reveals that genes up-regulated in older stages were generally already up-regulated in earlier stages (Figure 3B-D). For example the 10 up-regulated genes in F3 or six genes up-regulated genes in S2 were already up-regulated in F2 and S1 respectively when compared to F1 (Figure 3B-D). Similarly, genes down-regulated in earlier stages are also generally down-regulated in older stages when compared to F1. These observations highlighted an expression coherence from the F1 to F3 and S1 to S2 stages. The only exception was again between the F3 and S1 stages where a new set of genes is apparently activated with 18 of the total 27 up-regulated genes not having been up-regulated in the F3 stage (Figure 3C). Amongst these S1 newly activated genes *AtPrx03*, *AtPrx10, AtPrx11, AtPrx25, AtPrx34, AtPrx46 *showed a fold-difference higher than 3.5 when compared to F1.

**Figure 4:**
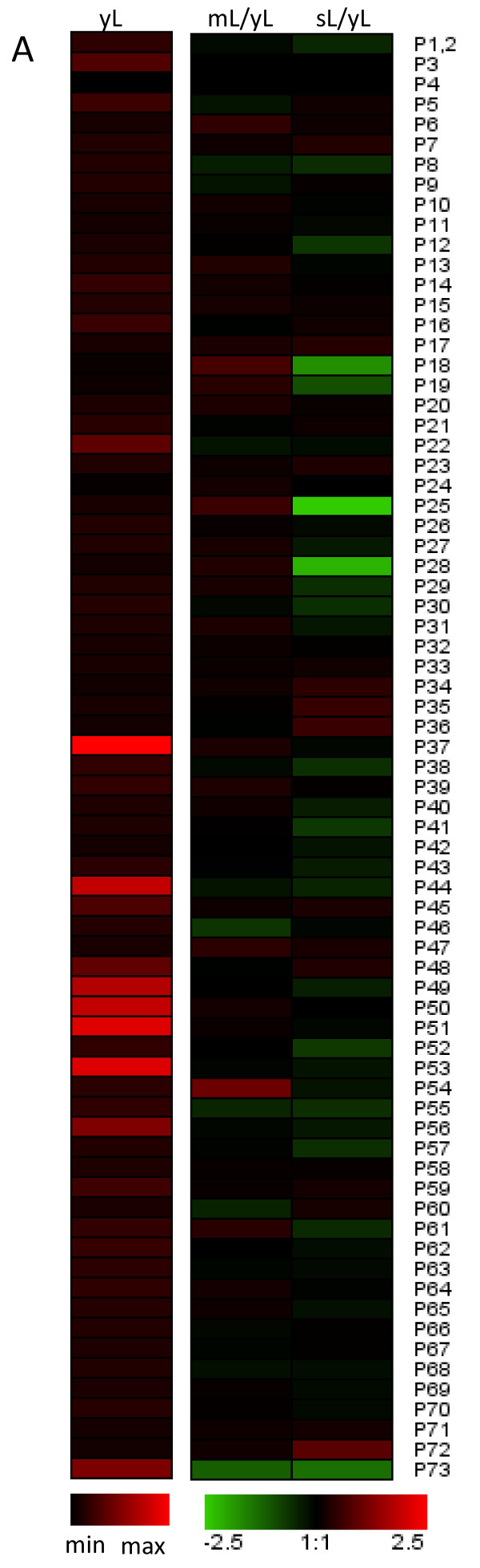
**Peroxidase expression profiles during leaf development at the gene level**. Macroarrays of young leaves (yL), mature leaves (mL), and senescing leaves (sL). In the first column expression level of each gene is represented as a ratio to the expression level of histone constitutively expressed gene (n = 3). In other columns, expression of peroxidase genes is represented as a ratio to their own expression level in yL stage in log scale.

Macroarray analysis performed on 3 stages of leaves (young, mature and senescening; Figure 4) determined (4-fold cut-off) that *AtPrx04*, *AtPrx10*, *AtPrx24 *and *AtPrx42 *have a significantly higher expression in flowers than leaves. On the contrary *AtPrx11*, *AtPrx15*, *AtPrx21, AtPrx22*, *AtPrx37*, *AtPrx50, AtPrx51*, *AtPrx53*, and *AtPrx59 *are more expressed in leaves than flowers. The comparison also demonstrated the specific genes induced or repressed in leaves and flowers over time. For example, the expression of *AtPrx59 *or *Atprx67 *increased during development of flowers and siliques whereas they were relatively stable in leaves. On the contrary, *AtPrx06*, *AtPrx07 *were up-regulated, and *AtPrx38*, *AtPrx41*, *AtPrx44*, *AtPrx73 *were down-regulated in all organs.

### Peroxidase isoforms pattern at different developmental stages

**Figure 5:**
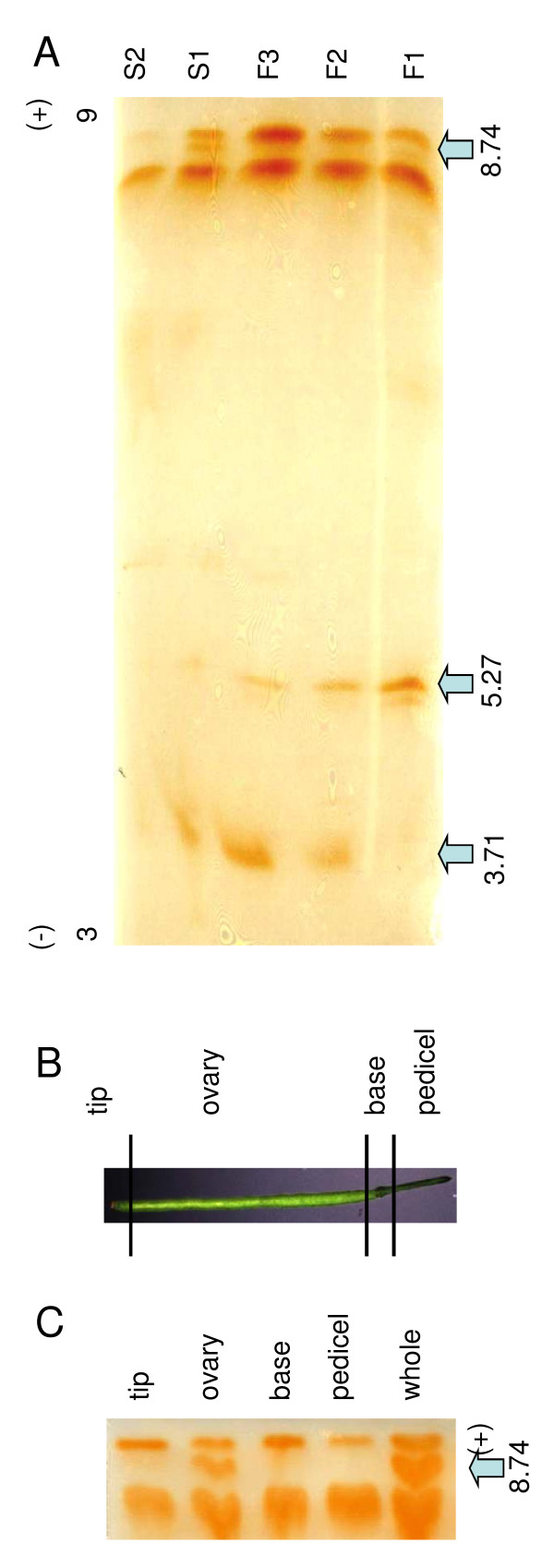
**Peroxidase expression profiles during flower and silique development at the protein level (A, C).** Isoelectric focusing (IEF) separation (pH 3.0-9.0) of isoperoxidases extracted from flowers and siliques various development stages (A) and from various zones of mature silique S1 (B, C). The gels were stained by o-dianisidine/H_2_O_2_. The arrows indicate the most significant band differences between development stages. The numbers indicate the isoelectric point of those bands (F1 = stage 1-12, F2 = stage 13-14, F3 = stage 15-16, S1 = stage 17, S2 = stage 18).

The pattern of the class III peroxidase isoforms at different flower and silique development stages was then analyzed by isoelectric focusing gel (IEF; Figure 5A). A maximum of 6 distinct bands were visible on the IEF in the different stages. Two basic bands (pI 8.3 and 9) were visible in all development stages although with great variation in intensity. These two bands showed the highest intensity in development stage F3. In development stage S2, the band of pI 8.3 was weaker than in younger stages and the band of pI 9 showed a very low intensity. A band of pI 5.18 was only found in F1. Another band of pI 5.27 decreased from F1 to S1 and disappeared in S2. However, a band of pI 3.71 was induced in F2 and increased over time to S1. Another band of pI 8.74 was only induced in S1. Siliques S1 were further cut in four samples (Figure 5B): the tip (the stigma and style), the ovary, the base (floral abscission zone), and the pedicel. The band of pI 8.74 was only detected in the ovary part (Figure 5C). In conclusion, different specific isoforms were clearly induced or repressed from F1 to S2.

### Class III peroxidase activity is localized in specific tissues in siliques

**Figure 6:**
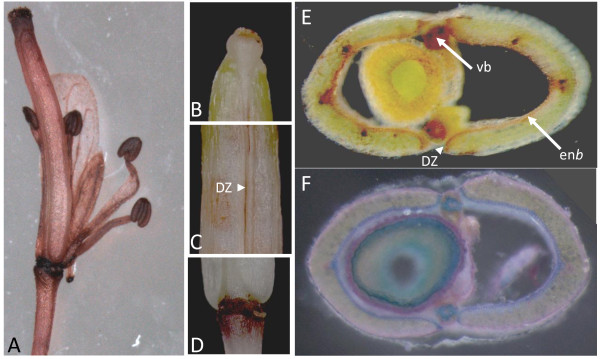
**Flower (A) and mature silique (B-E) stained *in situ *by guaiacol/hydrogen peroxide for peroxidase activity and by carmino green for lignin accumulation (F)**. Silique tip (B), silique valves and replum showing staining in the dehiscence zone (C), floral abscission zone (D) and transversal cuttings showing staining of the endocarp*b *layer and vascular bundles (E, F). DZ = dehiscence zone, en*b *= endocarp b, vb = vascular bundle.

In order to see if we could link class III peroxidase activity with specific tissues we stained the flower and siliques for class III peroxidase activity. Activity was localized in the stigma, anthers, and floral abscission zone of flowers (Figure 6A). It was also observed in the stigma, floral abscission zone and on valve margin DZ of siliques (Figure 6B-D). Transversal cuttings of siliques presented peroxidase activity staining in the vascular bundles and also in the en*b *(Figure 6E). Both tissues are highly lignified in siliques (Figure 6F).

Since class III peroxidase activity had been observed in the DZ and en*b *specialized cell layers (Figure 6) both known to be involved in pod shatter mechanism [17], we wanted to determine if class III peroxidase genes were involved in such a crucial mechanism.

### Treatment with a class III peroxidase inhibitor reduces lignin content of siliques and results in pod shatter delay

**Figure 7:**
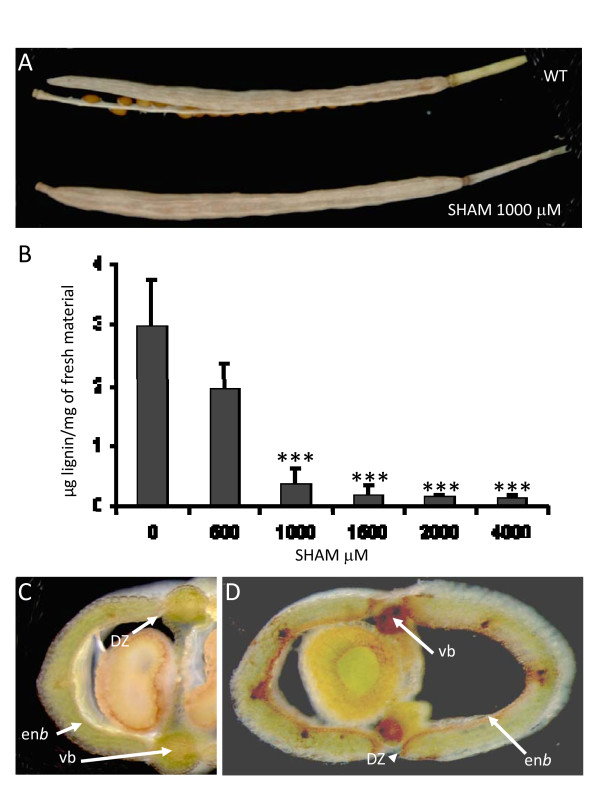
**A peroxidase inhibitor-treatment (SHAM) resulted in a delay in pod shatter (A), a lower lignin content of siliques S2 (B), and lower peroxidase activity (C) when compared to WT (D)**. Total lignin content of siliques S2 in plants treated with various concentration of SHAM (Student *t*-test p < 0.001, n = 6) (B). Transversal sections of siliques of plants treated with 1000 μM SHAM (C) and WT (D) stained *in situ *by guaiacol/hydrogen peroxide for peroxidase activity (DZ = dehiscence zone, en*b *= endocarp b, vb = vascular bundle).

We first treated bolting plants with a class III peroxidase inhibitor salicylhydroxamate (SHAM) and monitored silique maturation. Treatment of plants with SHAM resulted in a delay in pod shatter, in the 1000 μM and higher treatments (Figure 7A), and lowered total lignin content of siliques compared to non-treated plants (Figure 7B). A closer observation revealed that siliques opened easily when touched, indicating that hydrolysis of the DZ was unaffected but rather the spring-loaded mechanism was affected. Indeed it is thought that the lignification of the en*b *layer creates tensions within the pod [14]. A lower total peroxidase activity (Figure 7C) due to the inhibitor treatment resulted in a less lignified silique that consequently does not shatter normally when drying.

### Several peroxidase genes are regulated by shatterproof and related transcription factors

**Figure 8:**
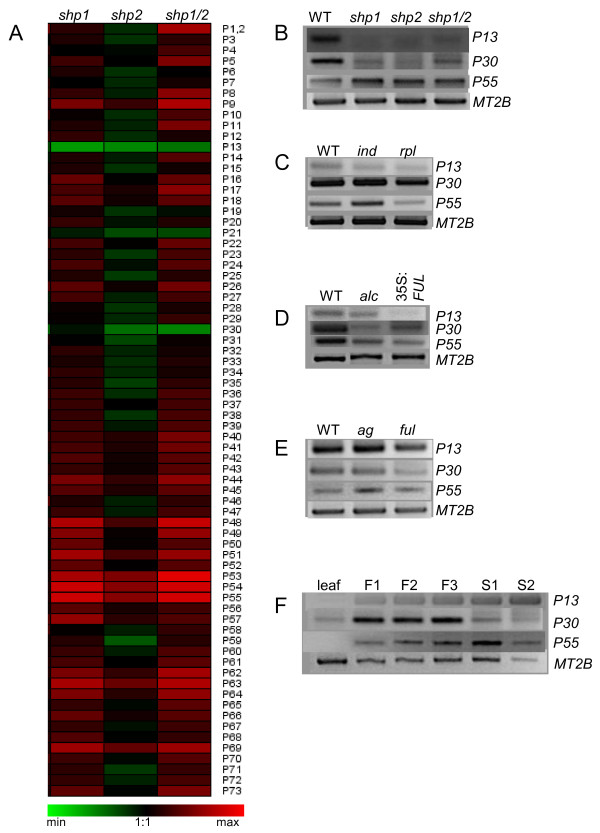
**Macroarrays (A) and semi-quantitative RT-PCR (B-G) in flower buds F1 in various mutant backgroung (n = 3)**. Expression of peroxidase genes in simple and double *shp *mutants is represented as a ratio to their own expression level in wild type (WT) (A). *AtPrx13 *(P13), *AtPrx30 *(P30), *AtPrx55 *(P55), and loading control (*MT2B*) expression level in shatterproof (*shp*) (B), indehiscent (*ind*), replumless (*rpl*) (C), alcatraz (*alc*), overexpressing fruitful (35S:FUL) (D), in agamous (*ag*), fruitful (*ful*) (E) lost-of-function lines, and in various organs and flower/silique development stages (F). (F1 = stage 1-12, F2 = stage 13-14, F3 = stage 15-16, S1 = stage 17, S2 = stage 18).

To identify specific peroxidase genes involved in pod shattering, we analyzed pod shatter mutant lines related to DZ lignification. Shatterproof transcription factors SHP1 and SHP2 are MADS-box transcription factors involved in dehiscence of the silique and control lignification of the siliques [17]. Because numerous peroxidase promoter regions contain a MADS-BOX binding sequence (data not shown), we chose to perform a transcriptomic study by macroarray analysis with *shp1*, *shp2 *and *shp1 shp2 *loss-of-function mutants, to identify class III peroxidase genes potentially regulated by shatterproof transcription factors. Macroarrays results (Figure 8A) were verified by semi-quantitative RT-PCR (Figure 8B). We focused on genes showing a significant altered expression in the three shatterproof mutant lines, that were in addition highly expressed in flowers/siliques when compared to leaves (see above). Three genes of interest have been identified with these criteria: *AtPrx13 *(0.64 ± 0.03, 0.67 ± 0.04 0.69 ± 0.04), *AtPrx30 *(0.38 ± 3, 0.32 ± 0.03, 0.50 ± 0.09) that were significantly down-regulated, and *AtPrx55 *(2.78 ± 0.31, 2.38 ± 0.26, 1.97 ± 0.24) that was significantly up-regulated in *shp1*, *shp2 *and *shp1 shp 2 *respectively when compared to their respective expression level in Col 0 background.

**Figure 9:**
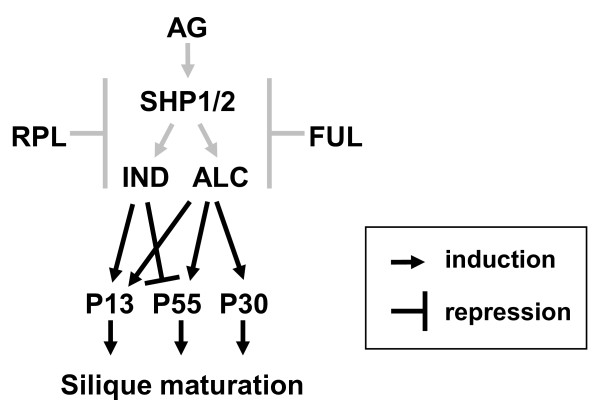
**Model of the observed interactions between the three identified peroxidases genes and the transcription factors known to be involved in pod shatter mechanism**. Interactions represented in grey are based on the literature.

To have a better idea of their regulation, we further monitored the expression of *AtPrx13*, *AtPrx30 *and *AtPrx55 *in a panel of other mutants of transcription factors involved in the regulation pod shatter (AG, FUL, RPL, IND and ALC; [16]) (Figure 8C-E). The results encompassing this study are schematized in the model presented in Figure 9 and are commented in the discussion section.

In order to determine the putative *in planta *function of AtPrx13, AtPrx30 and AtPrx55, we ordered the respective SALK T-DNA insertion mutant lines. Unfortunately no reduction of the corresponding transcript was observed in these lines (data not shown). The T-DNA was inserted in the promoter region for *AtPrx13 *(SALK_100989) and *AtPrx30 *(Garlic_102_A09.b.1a.Lb3Fa), and not at the predicted position for *AtPrx55 *(SALK_102284; data not shown). We further monitored the expression of *AtPrx13*, *AtPrx30 *and *AtPrx55 *in various organ and development stages by semi-quantitative RT-PCR (Figure 8F). *AtPrx13 *and *AtPrx55 *were mainly expressed in silique stages. *AtPrx30 *was mainly expressed in flower stages. However the promoter regions of these three genes were assessed by PLACE (Table 4). Promoters of the three genes showed some similar regulatory sequences. For example GA-responsive elements, but also water-stress and light regulated elements (data not shown) were found in all of them. *AtPrx13 *and *AtPrx55 *also contained a cis-sequence CARGAT found in promoter region of MADS-box flowering-time genes. In addition *AtPrx30 *and *AtPrx55 *showed an AGL2 (SEPALLATA1) binding sequence, whereas *AtPrx30 *showed a WUS binding site. It is interesting to note that AGL2 binding sequence was found in no other promoter region of class III peroxidase genes *AtPrx30 *and *AtPrx55*. CARGAT and WUS binding sequence were found in only 6 and 18 of those respectively. Several other elements involved in phytohormone responsiveness were also found: abscisic acid (*AtPrx13*, *AtPrx55*), auxin (*AtPrx13*, *AtPrx30*), cytokinin (*AtPrx30*, *AtPrx55*), ethylene (*AtPrx13*, *AtPrx55*) and salicylic acid (*AtPrx13*). Nevertheless these last motifs are found in many of the promoter regions of other class III peroxidases. Therefore the possibility that the motif is found simply by chance increases. However, at this point the specific function and precise role of *AtPrx13*, *AtPrx30 *and *AtPrx55 *remains intriguing.

**Table 4:** Selected cis-regulatory element present in the 1000 bp upstream of the *AtPrx13, AtPrx30, and AtPrx55 *genes and their frequency in all 73 class III peroxidase gene promoters obtained with PLACE database

			Position in gene promoter			
				
Motif	Sequence	Function	*AtPrx13*	*AtPrx30*	*AtPrx55*	Frequency (%)
**ABRE**	MACGYGB	abscisic acid responsiveness	251, 510(-)	n.f.	129	45
**AGL2**	NNWNCCA[W]_4_TRG[W]_2_AN	binding sequence of AGL2	10	n.f.	383(-)	3
**ARFAT**	TGTCTC	auxin response factor binding site	319	936	n.f.	34
**ASF1**	TGACG	activation of genes by auxin/salicylic acid	543	n.f.	n.f.	45
**CARGAT**	CC[W]_6_GG	found in MADS-box flowering-time gene	14	n.f.	386	11
**CPBCSPOR**	TATTAG	cytokinin-enhanced protein binding *in vitro*	n.f.	40, 287(-), 647(-)	518	68
**ERELEE4**	AWTTCAAA	ethylene responsive element	621(-)	n.f.	414(-), 555(-)	41
**GARE**	TAACAGA	gibberellin-responsive element	773	n.f.	n.f.	11
**GAHV**	TAACAAA	gibberellin response complex	n.f.	316	670(-), 814	59
**WUSTAG**	TTAATGG	target sequence of WUS	n.f.	917(-)	n.f.	26

Indicated positions are relative to the ATG codon (M = A/C, W = A/T, n.f. = not found).

## Discussion

### The increase of total class III peroxidase activity is due to only a few specific peroxidases

Class III peroxidase activity increased during development and senescence in different tissues of several plant species [18-21]. Class III peroxidases are known to be involved in several events taking place during development and maturation such as chrorophyll degradation, anthocyanin accumulation or lignification [22-24]. In the present study in accordance with the literature [25-27], anthocyanin and lignin content increased whereas chlorophyll content decreased while total peroxidase activity increased. Nevertheless a plethora of other functions have been described and will be certainly further linked in the future to peroxidase activity [7]. However the specific genes involved in the general peroxidase activity increase as well as their specific function have not been identified yet in *A. thaliana*.

In this study, macroarrays allowed analysing temporal gene expression of the 73 class III peroxidase genes during flower and silique developments in *A. thaliana*. The expression profiles of peroxidase genes varied during flower and silique developments. Our data indicated a general lower gene expression level in the silique development stages but also an induction of different set of genes during the transition from flower to silique development. We identified a number of genes that are, within flowers, specifically or predominantly expressed in one development stage and are probable components of the gene networks involved in floral organ development. Indeed some of the events taking place in each of the development stages can be linked with peroxidase activity and localisation.

Class III peroxidases can regulate directly or indirectly the cell wall architecture through their catalytic and hydroxylic cycles. In growth stage F1, *AtPrx33 *and *AtPrx43*, already identified by a proteomic approach as having a role in cell elongation of hypocotyls [28], are highly expressed. At stage F2, the stigma is receptive, anthesis take place and fertilization occurs. Previously a stigma-specific class III peroxidase gene, *SSP *(*stigma-specific peroxidase*) was identified which is expressed exclusively in the stigmas of an Asteraceae, *Senecio squalidus *L. [29,30]. Expression of *SSP *increases during flower development, to reach a maximum in newly opened flowers when stigmas are most receptive to pollen. So far its function is unknown but it may be related to regulation of H_2_O_2 _levels in stigmas. *AtPrx58 *appears in our study to have its highest level of expression in stage F2. In line with these data it has been identified as a stigma-specific gene and during pollen-pistil interaction by microarrays in two different studies [31,32]. In stage F3 sepals, petals and stamens wither and fall from the fruit. *AtPrx51 *that is highly expressed in this stage according to our study, has also been identified during stamen abscission by microarrays [33]. However, the floral abscission zone is also strongly stained *in situ *for peroxidase activity and suberin (unpublished data). Cross-linking of the phenolic monomers in the formation of suberin has been linked with peroxidase activity [4]. Peroxidases could have a function in defense of the floral abscission zone against biotic attack, either through a cell wall cross-linking activity (formation of lignin, extensin cross-links, dityrosine bonds; [5]) or by creating a highly toxic environment by producing ROS [34,35], which results in adverse growth conditions for microorganisms. A good candidate for such function could be *AtPrx50 *for example. Indeed this gene is highly expressed during F3 stage in our study and has been identified during various studies concerning stresses [36,37] as well as stamen abscission [33]. During stage S1, the fruit elongates to protect the seeds throughout their development. In addition, the valve margin and en*b *layer lignified. Growth and lignification are functions classically attributed to class III peroxidases [22]. *AtPrx02 *has been shown to be involved in lignification, *AtPrx34 *in root growth, and *AtPrx45 *in cell elongation [28,38,39]. At the end of fruit development at stage S2 the fruit yellows. Chlorophyll breakdown has been linked to peroxidase activity [23]. Nevertheless none of the genes expressed at this stage has been related yet to such a function.

To evaluate our data we compared our results with other published studies. Another study using the same macroarrays has been done earlier on inflorescences [13]. When comparing the two works, there was more convergence between highly expressed genes (8 out of 10) than lowly expressed genes (3 out of 10). A major difference was found for *AtPrx16 *that is reported as lowly expressed in Valério et al. (2004) study and is amongst the most expressed genes in our study. Other authors performed whole genome microarray analysis on various flower stages [40-43]. Nevertheless, none of these studies has been performed on mature and senescent siliques limiting comparisons with our work. Four of the genes (*AtPrx03*, *AtPrx40*, *AtPrx42*, and *AtPrx63*) that we found as highly expressed in flower buds F1 were also found as highly expressed in at least one of the other microarray studies. Only one gene (*AtPrx07*) that we found as expressed at low amounts was also found as lowly expressed in one other study [40]. However, when comparing the 10 lowest and 10 highest expressed genes in flower buds only 2 and 7 genes coincided respectively between the two microarray analysis [40,43]. This clearly suggested that the highest expression levels are more reliably monitored than the lowest expression levels with these array techniques, including macroarrays and microarrays. Other techniques (e.g. RNA-seq) need to be used to study low-expressed genes. However it has to be noted that class III peroxidases have homologies ranging from 28% to 98% at the nucleotide level [13]. The advantage of our home-made macroarrays is that we used a set of primers exhibiting a maximum of only 70% homology with any other sequence of the *A. thaliana *genome [13]. Such an approach was of course not possible with whole genome microarrays such as those used by other authors [41-43], since they examined thousands of genes. It can therefore not be excluded that these authors detect an unspecific peroxidase genes expression level due to some level of cross-hybridation [13]. However, differences between our results and those of other scientists can be attributed to biological variation, growth conditions, experimental variations and use of different detection criteria. The use of a dedicated macroarray rather than a commercially available microarray also allowed analyzing data on genes not present on microarrays, such as *AtPrx13*. However, although these array studies are of great value, they provide information that need to be confirmed.

We therefore continued the study at the protein level. On IEF a modification of the pattern of peroxidase isoforms between different development stages was also observed. In addition, different expression patterns were observed between the different parts of the S1 siliques: the band of pI 8.74 was only present in the ovary. Nevertheless, only six major bands were visible on the IEF, indicating that from the 73 peroxidase genes only the more active are visible and/or also that one band-particularly the thicker ones-might be formed by several peroxidases of close pI. However, different specific isoforms were clearly induced or repressed in plant organs and probably as well in specific tissues and cell types over time. Our data indicated a general lower number of peroxidase isoforms in the silique S2 development stages. This result together with the macroarray analysis confirmed that the increase of total peroxidase activity observed with development in flower and siliques was due to only a few specific genes and not to an increase of the expression of all peroxidase genes. Senescence is known to be characterized by a progressive decrease of total protein content [44]. Probably reduced synthesis and enhanced proteolysis are both responsible for protein loss observed during senescence. In this regard, synthesis of all thylakoid proteins is known to be severely curtailed in senescing bean leaves except for the D1 protein of photosystem 2 [45]. The results presented in our study suggest that in the case of peroxidase a reduced synthesis might be predominant for the vast majority of the genes, therefore further supporting the existence of a functional specialization of peroxidases.

### Peroxidases are involved in pod shatter and probably other cell separation processes

Presence of peroxidase activity has been previously reported in stigma, anthers and AZ from different plants [30,46,47], but to our knowledge it is the first time that peroxidases activity was localized in the en*b *and DZ. However, a common feature of several of these areas where peroxidases have been observed is their involvement in some kind of cell separation process including pod shatter, anthers dehiscence, and floral organ abscission [48-50].

To further identify the genes potentially expressed in en*b *and DZ, we analyzed class III peroxidase gene expression profiles in mutants related to pod-shattering and compared to their expression in WT. *AtPrx13 *and *AtPrx30 *were significantly down-regulated, on the contrary *AtPrx55 *was significantly up-regulated in the three loss-of-function *shp *mutant lines, suggesting that these three genes are expressed in en*b*. The three genes were mainly expressed in flower or siliques. In addition, several regulatory sequences identified in the promoter regions of these three genes further supported their role in flower development. The expression of *AtPrx13*, *Atprx30 *and *AtPrx55 *were also monitored in a panel of other loss-of-function mutants related to en*b *development (*ag*, *ful*, *rpl*, *ind *and *alc*). FUL, IND, ALC, SHP1/2 appear to be expressed together in the en*b *layer, although the nature of the interaction of these antagonistic factors is unclear and their precise role in en*b *development has remained elusive [16,17]. However the loss of en*b *lignification is only seen in the *ful ind alc shp1 shp2 *quintuple mutant, indicating that all genes are involved in en*b *lignification. In our model (Figure 9) it can be observed that *AtPrx55 *expression level is affected in all these mutant lines, indicating a good candidate for lignification of siliques except that the gene was down-regulated by SHP1 and SHP2. Indeed, we would expect the opposite regulation for a gene involved in lignification of en*b*. However, class III peroxidases can generate highly reactive ROS which can possess an intrinsic activity, or can act as part of signal transduction pathways [51,52]. Maybe AtPrx55 is one of the peroxidases with such activity. Unfortunately, AtPrx55 is not documented in the literature preventing further hypothesis on its putative function. *AtPrx30 *on the other hand is up regulated by the SHP transcription factors and has also been identified by microarrays in stamen AZ and in monolignol polymerization suggesting its putative involvement in both silique DZ as well as stamen AZ lignification [33,38]. All cell separation process involves the differentiation of specialized cell types and a tight co-ordination of molecular and biochemical events [53]. There is accumulating evidences that common mechanisms exist between the different cell separation process [48,49,54]. For instance RDPG1, an endo-polygalacturonase involved in cell wall breakdown during silique opening of oilseed rape (*Brassica napus*), has been found also in dehiscence zones of anthers and floral abscission zones and stylar tissues during pollen tube growth in *Arabidopsis *and *Brassica *[48]. Moreover transcription factors (ALC, AG) known to be involved in regulation of pod shattering have also been identified in a microarray study concerning stamen AZ [33]. In addition, several peroxidase genes (*AtPrx03*, *AtPrx17*, *AtPrx21*, *AtPrx31*, *AtPrx33*, *AtPrx34*, *AtPrx42*, *AtPrx45*, *AtPrx50*, *AtPrx51*, *AtPrx52*, *AtPrx53*, *AtPrx67*, *AtPrx71*) have been identified in the study concerning stamen AZ, illustrating the redundancy of this protein family and the complexity in assigning a function to a class III peroxidase genes. Six of these genes (*AtPrx21*, *AtPrx31*, *AtPrx33*, *AtPrx34*, *AtPrx53*, *AtPrx71*) were identified in our study on *shp *loss-of-function mutants, further supporting the existence of common mechanisms in the various cell separation processes. Several of these genes have also been reported in studies concerning responses to abiotic or biotic stresses (*AtPrx03, AtPrx21, AtPrx33, AtPrx34, AtPrx45, AtPrx50, AtPrx52, AtPrx67, AtPrx71*) or lignin synthesis (*AtPrx17*, *AtPrx53*) giving some additional indication on their putative role [7,35-38,55-61]. *AtPrx13 *on the opposite is not documented in the literature and in the microarray databases. However, the precise role of each peroxidase gene in cell separation processes needs to be elucidated.

In *A. thaliana*, the DZ and the en*b *are composed by highly specialized cells essentially involved in the pod shatter mechanism. A lignification of the en*b *layer happens at stage 17 of silique development, and is necessary for a proper shatter mechanism [14,16]. A well known function of class III peroxidases is lignification [22]. In our study, the observation of peroxidase activity in these specialized tissues further supported a possible involvement through lignification of various peroxidase genes in cell separation processes and particularly in the pod shattering mechanism. In the present study we showed that plants treated with peroxidase inhibitor produced siliques with lower lignin content resulting in a delay of pod shattering. A good spatio-temporal correlation was found between abscission zone weakening and increased peroxidase activity in *Phaseolus*, cherry fruit, cotton, and *Citrus *[47,62-65]. Nevertheless several mechanisms that can be related to peroxidase activity have been observed in the abscission zone. Expression data associated with the leaf abscission in *Citrus *indicated the occurrence of a double defensive strategy mediated by the activation of a biochemical program including ROS scavenging, defense and PR genes, and a physical response mostly based on lignin/suberin deposition [65]. ROS scavenging, defense and lignin deposition are linked to peroxidases and may be related to the considerable increase in peroxidase activity [47,66]. Lignin has been proposed to have a double role as a physical barrier of the protective layer developed on the part of the organ that remains attached to the plant and also as component of the fracture line favoring cell separation [65]. On the other hand class III peroxidases can also produce ROS [34,35], which results in adverse growth conditions for microorganisms. During cell separation processes, peroxidase expression could also be triggered as a preventive defense mechanism against pathogen attacks [67].

## Conclusions

Our data illustrate the difficulty in interpreting total peroxidase activity data when trying to link it to specific functions. During flower and siliques development total peroxidase activity increased sharply, but the level of expression of most genes and the number of bands visible on IEF stayed stable or even decreased. This study confirms the necessity to study peroxidases individually to comprehend the roles of the class III peroxidase family. Indeed, many functions have been attributed to class III peroxidases [3], however the majority of individual peroxidase genes function still needs to be assessed [7]. The approach used in this study - to identify class III peroxidase genes changes specific to flower and silique maturation in *A. thaliana *by transcriptome analysis-highlights which are the key individual genes to investigate further by reverse genetics approaches. Whole transcriptome analysis is indeed the most promising approach for overcoming the difficulties associated with the study of class III peroxidase function. The dedicated macroarray used here proved to be a cheap and efficient tool for this aim.

## Methods

### Plant material and growth conditions

*Arabidopsis thaliana *plants were grown at 24°C under 16 h light/8 h dark and 60% humidity and with 80 μEinstein m^-2 ^s^-1 ^of light intensity first 7 days on ½ MS medium [68] and then transferred to soil. Organs such as flower buds (F1 = stage 1-12, see [16]), mature flowers (F2 = stage 13-14), senescing flowers (F3 = stage 15-16), mature siliques (S1 = stage 17), senescing siliques (S2 = stage 18), young leaves (yL = small leaves on top of rosette), mature leaves (mL = full expanded leaves) and senescing leaves (sL = partly yellowing leaves) used for total RNA and protein extractions were collected and immediately frozen in liquid nitrogen.

Ecotypes and mutants of *Arabidopsis *used were Col 0 (WT), L*er *(WT), *shp1-1*, *shp2-2*, *shp1-*1 and *shp2-2*, *AtPrx13 *(SALK_100989), *AtPrx30 *(Garlic_102_A09.b.1a.Lb3Fa), *AtPrx55 *(SALK_102284) in Col background (obtained from the European *Arabidopsis *Stock Centre [NASC], University of Nottingham, United Kingdom), *ful*, *ag *L*er*, (NASC), *alc-1*, *rpl-2*, *ind-2*, 35S:*FUL *(Col, gift from Lars Østergaard, John Innes Centre, Norwich, UK).

### Chlorophyll content

Fresh material was incubated overnight in the dark in 1 ml 80% acetone. Absorbance of the supernatant was measured at 663 nm and 647 nm and converted to μg of chlorophyll using the following formula:

$$\left({\text{DO}}_{\text{663nm}}\text{*7}.\text{15}\right)+\left({\text{DO}}_{\text{647nm}}*\text{18}.\text{71}\right).$$

### Anthocyanin content

Fresh material was ground in 300 μl 1% methanol-HCl and incubated overnight at room temperature. 200 μl water and 500 μl chloroform were added, and the insoluble residues were eliminated by centrifugation. The absorbance was measured at 530 nm and 657 nm and converted to μg of anthocyanin using the following formula:

$${\text{DO}}_{\text{53}0\text{nm}}\;-\left({\text{DO}}_{\text{657nm}}*0.\text{25}\right).$$

### Lignin content

Thioglycolic acid extraction of lignin was performed as described elsewhere [69] with some modifications. Fresh material was resuspended twice in 1 ml ethanol. After centrifugation at 10000 g, the pellets were dried at 60°C. The insoluble cell wall material (about 20 mg) was resuspended in 500 μl 2 N HCl and 50 μl thioglycolic acid (Fluka). The mixture was heated for 4 h in boiling water and centrifuged at 10000 g for 15 min. After washing with water, the pellets were resuspended in 500 μl 0.5 N NaOH and left 18 h shaking softly at room temperature. The insoluble residues were eliminated by centrifugation and lignin thioglycolate was precipitated by the addition of 100 μl concentrated HCl (4 h at 4°C). After centrifugation at 10000 g for 15 min, the pellets were resuspended in 1 ml 0.5 N NaOH. The absorbance was measured at 280 nm and converted to μg lignin using lignin alkali (Aldrich) as a standard.

### Macroarray experiments

Total RNA was isolated from flowers or leaves with Tri-reagent solution (Sigma) and from siliques according to a protocol described elsewhere [70] with the addition of a sodium acetate wash to remove excess polysaccharides. Messenger RNA for the cDNA probe synthesis was obtained from 500 μg total RNA with the PolyAtract mRNA Isolation System kit (Promega). The cDNA was labeled by incorporating 50 μCi of [alpha-^32^P]dATP during reverse transcription using random primers, according to the ImPromII RT (Promega) protocol.

PCR products (90-400 bp) corresponding to the 73 peroxidases genes and two pseudogenes were used for the macroarray approach. The specificity of amplicons is related to their low homology (less than 70% against the whole *Arabidopsis *genome) and has already been verified experimentally [13]. 50 ng of each amplicon were blotted on a nylon membrane and hybridized in triplicates with the radiolabeled cDNA library as described previously [13]. The constitutively expressed *A. thaliana *putative histone H4 (At2 g28740) was used as positive control to normalize the expression data and the peroxidase pseudogene I was utilized as a negative control. Genesis software was use to graphically present the ratio of expression level in log scale [71]. Only genes showing a differential expression of at least 1.3 up or 0.7 down and a significant Student t-test score (p < 0.05) were included in Venn diagrams.

### Relative-quantitative Reverse Transcriptase PCR

Reverse Transcriptase-PCR (RT-PCR) was used as a semi-quantitative method to assess the expression of peroxidase genes. Flowers or siliques were harvested and frozen immediately in liquid nitrogen. 100 mg of tissue sample were ground in liquid nitrogen, and total RNA was extracted with the Tri-reagent solution (Sigma) according to the instructions of the manufacturer. After quantification of the concentration by spectrophotometry and confirmation by electrophoresis, 1 μg of the crude RNA preparations was treated with one unit of RNase-free DNase I (Promega). The DNA-free RNA was then used as a template during reverse transcription according to the ImPromII RT protocol from Promega. PCR amplification was conducted for up to 35 cycles using the following thermal profile: denaturation at 95°C for 1 min, annealing at 55°C for 1 min, and polymerization at 72°C for 30 s, with a 10 min terminal extension step at 72°C. Primers used for peroxidases genes were the same as for the macroarray experiments [13]. Reactions without RT were used as control to rule out contamination by genomic DNA. To determine whether comparable amounts of RNA from the different tissues had been used for RT-PCR, the level of metallothionein gene, *MT2B *(5'-ACATGTCTTGCTGTGGTGGA-3' and 5'-ATGACCAAACCATAAAAAC ACAC-3') was used as an internal standard [72]. Densitometric analysis of ethidium bromide-stained agarose gels (1%) was then performed. The relative abundance of the transcript within the samples was calculated as the ratio of the intensities of the gene amplicon to the *MT2B *amplicon. Reactions were performed in triplicate and averaged.

### Separation of class III peroxidase isoforms

Soluble proteins were extracted from the various organs by grinding in 20 mM Hepes, pH 7.0, containing 1 mM EGTA, 10 mM vitamin C, and PVP PolyclarAT (100 mg/g fresh weight). The extract was centrifuged twice for 10 min at 10000 g. Each extract was assayed for proteins levels with Bio-Rad assay (Bio-Rad) and for total class III peroxidase activity using guaïacol/H_2_O_2_.

Gel isoelectric focusing (IEF) was performed with Servalyt precotes gels pH 3-10 (Serva) according to the instruction of the manufacturer. 10 μg of total proteins were loaded per line. Class III peroxidase activity was revealed with o-dianisidine/H_2_O_2_.

### Organs staining

After 12 h of bleaching with 100% EtOH to remove chlorophyll, flowers and siliques were stained for class III peroxidase activity using guaiacol/H_2_O_2 _resulting in an orange to brown coloration. Lignin localization was performed with green carmino that colours lignified tissues in green and cellulose in pink. Organs were incubated 10 min in the different preparations. Reactions were stopped by transferring the organs in distilled water.

Except when specified, observations were made with a MZ 16 Leica stereomicroscope (Leica Mycrosystems GmbH). Pictures were taken with a DC300F Leica camera. Settings were identical for all the pictures in an experiment. Each kind of experiment was repeated at least 4 times with similar results.

### Salicylhydroxamate (SHAM) treatment

*Arabidopsis thaliana *seeds were germinated on ½MS medium. Four one-week-old seedlings were transferred to a 1000 mL pots filled with modified one-quarter-strength Hoagland nutrient solution (Sigma) supplemented with 20 μM Fe-HBED (Strem Chemical). 500 to 4000 μM SHAM - a class III peroxidase inhibitor [73]- was added in nutrient solution just after bolting. Three pots per treatment were set up. The nutrient solution was renewed every week and aerated continuously.

## Authors' contributions

CC designed, performed and wrote up the work. CD developed the class III specific macroarrays, participated in the design and coordination of the study and helped to draft the manuscript. Both authors read and approved the final manuscript.
